# The Antioxidant Resveratrol Protects against Chondrocyte Apoptosis by Regulating the COX-2/NF-*κ*B Pathway in Created Temporomandibular Osteoarthritis

**DOI:** 10.1155/2021/9978651

**Published:** 2021-07-09

**Authors:** Wen Li, Shiyu Hu, Xuepeng Chen, Jiejun Shi

**Affiliations:** Stomatology Hospital, School of Stomatology, Zhejiang University School of Medicine, Clinical Research Center for Oral Diseases of Zhejiang Province, Key Laboratory of Oral Biomedical Research of Zhejiang Province, Cancer Center of Zhejiang University, Hangzhou 310006, China

## Abstract

Temporomandibular joint osteoarthritis (TMJOA) is characterized by chronic inflammatory degradation of mandibular condylar cartilage (MCC). Studies have found a positive correlation between inflammation and cyclooxygenase- (COX-) 2 in OA pathology. NF-*κ*B is a crucial transcription factor of inflammatory and immune responses in the cause of TMJOA pathology. Resveratrol (RES) plays a critical role in antioxidation and anti-inflammation. But, studies on the effects of RES on TMJOA are very limited. So, the purpose of this study is to investigate the antioxidant and protective effects of RES against MCC degradation through downregulating COX-2/NF-*κ*B expression. In vitro studies, the MCC cells were divided into three groups: the NC group, OA group, and RES group. The optimum dose of RES (10 *μ*M) was determined. The TMJOA model of mice was created by injection of collagenase. And mice were injected with RES (100 *μ*g/10 *μ*l) 3 times one week for 4 weeks in the RES group. The expressions of COX-2, P65, MMP1, MMP13, COL2, and ACAN were measured by RT-PCR. Morphological changes of MCC were studied with HE staining. The results showed that inflammation could induce MCC degradation in vitro and vivo, while RES could reverse the degradation. Meanwhile, RES could downregulate COX-2/NF-*κ*B/MMP expression and increase cartilage markers in vitro and vivo studies. The results indicated that RES treatment had antioxidant effects against chondrocyte apoptosis by downregulating the COX-2/NF-*κ*B pathway in created TMJOA.

## 1. Introduction

Temporomandibular joint osteoarthritis (TMJOA) is characterized by chronic inflammatory degradation of mandibular condylar cartilage (MCC) [1, 2]. The exact etiology of TMJOA is still unknown, but several risk factors have been reported, such as oxidative stress and inflammation [3–5]. Many studies reported that inflammatory pathway as a “triggering factor” is closely related to TMJOA [6]. Our previous study showed that interleukin-1*β* (IL-1*β*) contributed to the pathogenesis of TMJOA and induced the inflammation and destruction of the MCC [4]. The nuclear factor kappa B (NF-*κ*B) family of transcription factors is a key regulator of immune development, immune responses, and inflammation [7, 8]. The association between NF-*κ*B and the pathogenesis of TMJOA has been confirmed in animal experiments [4]. IL-1*β*-activated NF-*κ*B promotes OA development via its action on MCC. Therefore, in order to alleviate or even cure TMJOA, it is of great significance to understand the molecular mechanism of the effect of inflammation on TMJOA.

Cyclooxygenase (COX) is the rate-limiting enzyme for arachidonic acid to synthesize prostaglandin E2 (PGE2)—one of the major mediators involved in the degradation of articular cartilage in OA [9]. There are two isoforms of COX in human that have been described, COX-1 and COX-2.COX-1 is expressed constitutively in various tissues for homeostasis maintenance, while COX-2 is induced by numerous stimuli including excessive mechanical stress, chemical stimuli, and inflammation, being regarded as a pathological enzyme [10, 11]. Studies have found a positive correlation between inflammation, oxidative stress reaction, and the expression of COX-2. Induced COX-2 expression leads to the secretion of proinflammatory cytokines such as PGE2, PGH2, and VEGF, inducing the generation of oxidative stress-associated products like oxygen radicals and ROS, and resulting in the cellular injury [12, 13]. And in TMJ, COX-2 plays an important part in condylar cartilage degeneration [14]. Therefore, targeting COX-2 may be a promising method to suppress TMJOA.

Resveratrol (RES) is a kind of multifunctional biological polyphenol and plays a critical role in the cell viability, proliferation, anti-inflammation, and antioxidant [15] properties, moreover, in the prevention and progression of chronic diseases related to inflammation [16]. Some studies have demonstrated that RES alleviates rheumatoid arthritis by reducing inflammation, inhibiting MAPK signaling pathways, and suppressing angiogenesis [17]. Although some studies revealed that the intra-articular RES treatment could exert a curative effect by preventing the inflammation and cartilage destruction of TMJOA [18], the mechanism remained unknown.

In this study, we investigated the therapeutic effect and the possible mechanism of RES on MCC in vitro and in OA mice, then presumed that RES has restorative effects on cartilage destruction by inhibition of COX-2/NF-*κ*B signaling pathways.

## 2. Materials and Methods

### 2.1. Cell Culture and RES Treatment

The procedures for cell culture were referred to Izawa's study [4]. Briefly, cells were isolated from condylar cartilage of 6-week-old female C57BL/6J mice by mechanical dissection and digestion with trypsin and collagenase II. All cells were cultured in CO_2_ incubator (DMEM, 37°C, 5% CO_2_). Third cultures were treated with 5, 10, 20, 50, and 100 *μ*M RES (Sigma, USA) for 12 h and then tested by MTT to observe the optimum dose of RES on cell viability.

### 2.2. IL-1*β*-Induced Apoptosis

The third-generation cells were divided into three groups: the normal control group (NC), osteoarthritis group (OA), and RES treatment group (RES). The OA group cells were induced by IL-1*β* (10 ng/ml, PeproTech Inc., USA) [19]. The RES group cells were treated with IL-1*β* (10 ng/ml) and RES (10 *μ*M). Cell apoptosis was measured by flow cytometry using AnnexinV Apoptosis Detection Kit I (BD Bioscience).

### 2.3. siRNA Treatment for COX-2

The cells (2.0 × 10^5^/well) were seeded and cultured in a 6-well culture plate until 70% confluence, the culture medium was changed to reduced serum medium (Opti-MEM, Gibco) for overnight, and then, the cells were treated with COX-2 siRNA (GenePharma, China), or control vehicle siRNA with lipofectamine reagent (Invitrogen) for 24 h, according to the manufacturer's instructions. Then, treated cells were cultured with IL-1*β* (10 ng/ml). 24 h later, the cells were harvested and used for western blot analysis.

### 2.4. Setup TMJOA Model

12 female mice (8 week old, 17.3 ± 1.5 g) were purchased from the Animal Center of Zhejiang Academy of Medical Sciences. The mice were divided into the normal control group (NC), OA group (OA), and RES group (RES). The TMJOA model was set up by injection of collagenase (12.5 *μ*l, Sigma Biochemical, St. Louis) in the upper cavity of TMJ according to previous studies and made some modifications [2, 18]. 1 week later, the OA mice were injected by RES (100 *μ*g/10 *μ*l) [18] 3 times one week. 4 weeks later, the mice were sacrificed and the condyle was harvested. Animals were treated according to the Guidelines for Animal Care at Zhejiang University School of Medicine at Hangzhou.

### 2.5. Histopathology

The condylar tissues were fixed in 4% PFA, then decalcified in 10% EDTA for 4 weeks. After dehydration, the samples were embedded in paraffin. For histological analysis, sections were deparaffinized and stained with hematoxylin and eosin (H&E). Mankin score was used to evaluate the structure changes of condylar articular cartilage.

.

### 2.6. Measurement of COX-2/P65/MMPs and Cartilage Markers

Total RNA was extracted with TRIzol (Invitrogen), and the cDNA was synthesized according to the GeneAmp PCR kit (ABI, USA). RT-PCR was performed according to the SYBR green RT-qPCR kit (TOYOBO Corporation). All PCR reactions were performed using iCycleriQTM (Bio-Rad, Hercules, CA, USA). The cycling conditions were 10 min at 95°C, followed by 40 cycles: denaturation at 94°C for 15 s, annealing for 30 s at 57°C, and extension at 72°C for 30 s. The primers of GAPDH, COX-2, P65, MMPs, and cartilage markers were shown in Table 1.

### 2.7. Statistical Analysis

SPSS 22.0 was used for the statistical analysis, and *P* value less than 0.05 was considered significantly different. Student's *t*-test was used for comparisons between two groups.

## 3. Results

### 3.1. Mechanism of COX-2 on MCC Degradation

**Figure 1:**
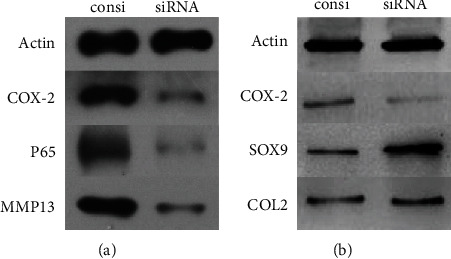
Effect of COX-2 on IL-1*β*-induced chondrocyte degradation. Chondrocytes treated with COX-2 siRNA or control vehicle siRNA were subjected to IL-1*β*. Whole-cell lysis was subjected to western blot analysis. (a) The effects of COX-2 on inflammation pathway. Inhibition of COX-2 could decrease the expression of P65 and MMP13. (b) The effects of COX-2 on MCC degradation. Inhibition of COX-2 could increase the expression of SOX9 and COL2.

To examine the effect and mechanism of COX-2 on cartilage degradation, chondrocytes were treated with COX-2 siRNA or control vehicle siRNA before being treated with IL-1*β*. P65, MMP13, and the cartilage markers (SOX9 and COL2) were measured by western blot. The present study found that COX-2 siRNA treatment could significantly decrease the expression of P65 and MMP13 and reverse the decrease of the expression of SOX9 and COL2 induced by IL-1*β* (Figure 1).

### 3.2. Effects of RES on IL-1*β*-Induced Apoptosis

**Figure 2:**
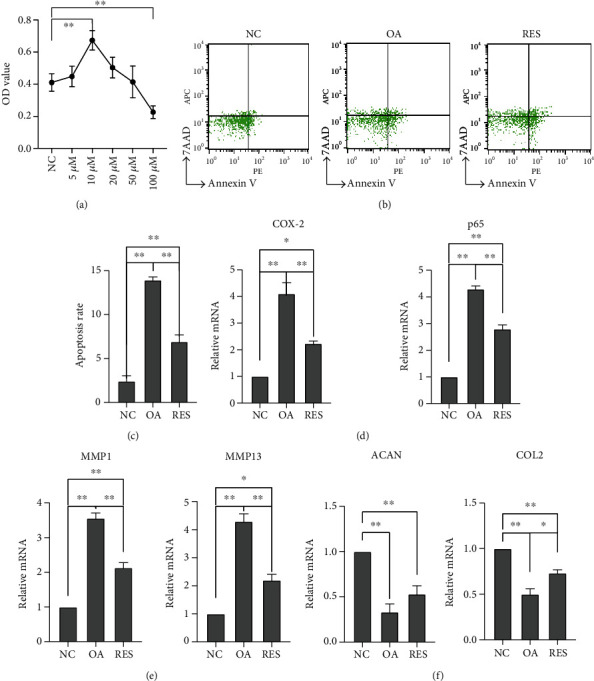
Effect of RES on IL-1*β*-induced apoptosis in the chondrocytes. (a) The optimum dose of RES was determined by MTT after cotreatment for 12 h. (b, c) Flow cytometric analysis for chondrocyte apoptosis. RES treatment could decrease cell apoptosis. (d) The mRNA expression of COX-2 and P65 in each group; RES treatment could suppress COX-2 and P65 expression in vitro. (e) The mRNA expression of MMP1 and MMP13 in each group. (f) The levels of cartilage markers' (ACAN, COL2) gene expressions in chondrocytes were determined by RT-PCR. RES treatment could increase cartilage markers' expression (*n* = 3; ^∗^*P* < 0.05, ^∗∗^*P* < 0.01).

The optimum dose of RES (10 *μ*M) was determined by MTT (Figure 2(a)). IL-1*β* induced the upregulation of apoptosis compared with the NC group. Meanwhile, RES could decrease the apoptosis induced by IL-1*β*(Figures 2(b) and 2(c)). The mRNA expression of COX-2, P65, and MMPs in the OA group increased significantly, and RES treatment reversed this increase (Figures 2(d) and 2(e)). The mRNA level of cartilage markers (COL2 and ACAN) decreased obviously in the OA group, and RES could increase the markers expression (Figure 2(f)).

### 3.3. Structure Changes of Condylar Articular Cartilage

**Figure 3:**
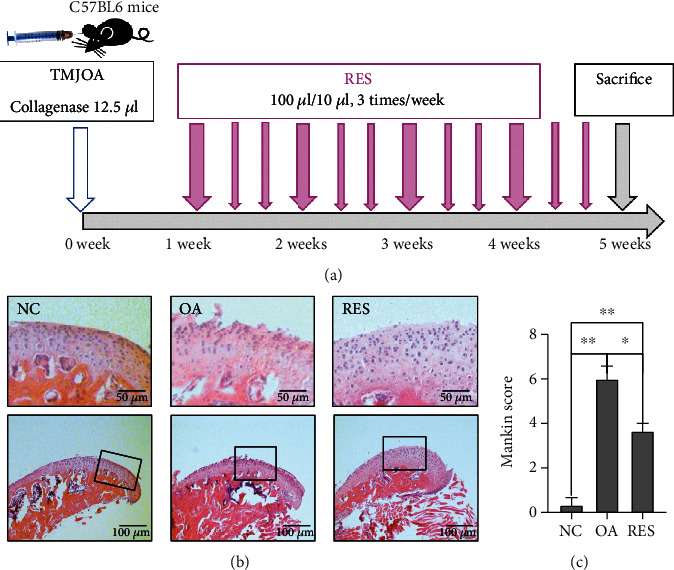
RES alleviated structure changes in mouse TMJOA model. (a) Procedure for setting up the TMJOA model and injecting RES. (b) Histological analysis of condylar cartilage by H&E staining. RES treatment could partly reverse cartilage degradation. (c) Mankin score of the condylar articular cartilage of three groups (*n* = 3; ^∗^*P* < 0.05, ^∗∗^*P* < 0.01).

HE staining showed normal morphology of joints in the normal control group, whereas the joints in the OA groups exhibited irregular surface. With intra-articular injection of RES, the surface of the articular cartilage was a little bit smoother than the OA group, and cartilage thickness was increased (Figure 3(b)). Mankin score revealed that RES could reverse the damage of MCC of OA mice to some extent (Figure 3(c)).

### 3.4. The Expression of COX-2/P65/MMPs and Cartilage Markers in MCC

**Figure 4:**
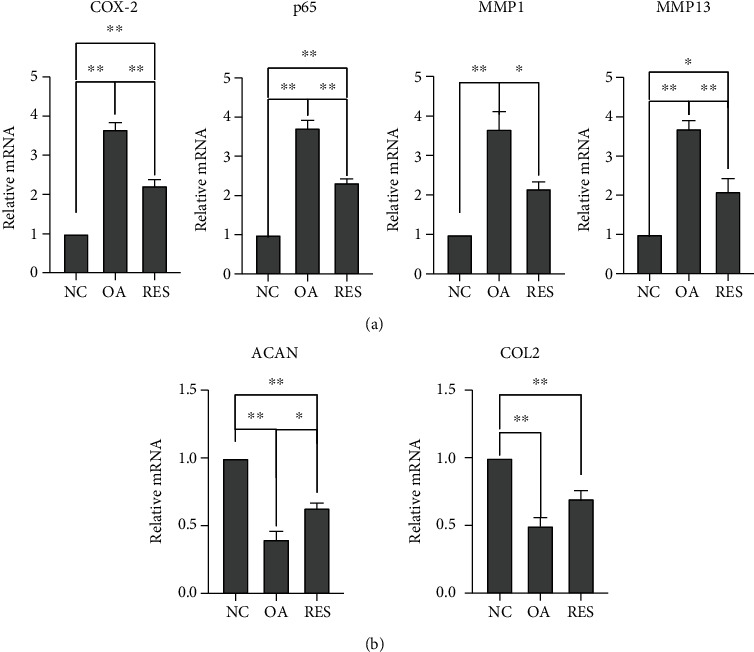
RES influenced the expression of COX-2/P65/MMPs and cartilage markers in mouse TMJOA model. (a) The mRNA expression of COX-2, P65, and MMP1 and MMP13 in MCC was detected by RT-PCR. RES treatment could suppress COX-2 expression and related inflammation pathway. (b) The levels of ACAN and COL2 gene expressions in the mouse condylar cartilage were determined by RT-PCR. RES treatment had therapeutic effects against MCC degradation (*n* = 3; ^∗^*P* < 0.05, ^∗∗^*P* < 0.01).

Compared with the NC group, the mRNA expression of COX-2, P65, and MMP1 and MMP13 increased significantly in OA mice, while the expressions of aggrecan and COL2 were significantly decreased in TMJOA mice. RES treatment significantly upregulated the gene expressions of cartilage markers and reduced the gene expressions of COX-2, P65, and MMPs (Figure 4).

## 4. Discussion

TMJOA is mainly characterized by the release of inflammatory cytokines, such as IL-1 and TNF-*α*, leading to the destruction of cartilage matrix. COX-2 is an important proinflammatory enzyme, whose abnormal expression is a significant marker of OA in joints [9, 14]. It is reported that normal human articular chondrocytes do not express measurable COX-2 mRNA, but inflammatory cytokines like IL-1 and TNF-*α* can induce articular chondrocytes expressing COX-2 mRNA [20, 21]. In TMJ, COX-2 plays an important part in condylar cartilage degeneration and progression of inflammation [5, 22]. When articular chondrocytes and synoviocytes from TMJ were treated with exogenous IL-1*β*, the expression levels of COX-2/PGE2 were enhanced [23, 24]. On the other hand, COX inhibitors decreased the expression of COX-2 and PGE2 production in condylar chondrocytes and fibroblast-like synoviocytes from TMJ, showing the anti-inflammatory effect of COX inhibitors [21, 22]. The present study found that IL-1 could increase the protein expression of COX-2 and decrease the expression of cartilage markers, while inhibition of COX-2 by siRNA could suppress the NF-*κ*B pathway by descending the expression of P65 and downstream factor MMP13. Meanwhile, inhibition of COX-2 could increase the expression of cartilage markers (SOX9 and COL2), which indicated that the COX-2 may be a potential therapeutic target against MCC degradation.

RES, which is known to have anti-inflammation and antioxidation effects, plays an important role in treating OA disease. And our study verified the therapeutic effect of RES on MCC cells by flow cytometry, which demonstrated that RES significantly reversed IL-1*β*-induced apoptosis in the chondrocytes. Some studies verified that RES exerted protective effects on OA through its anti-inflammatory property by the NF-*κ*B pathway [25, 26]. The present study agreed with previous studies, which revealed the anti-inflammation of RES on TMJOA by inhibiting the COX-2 gene expression, NF-*κ*B pathway, and downstream factors MMPs. MMPs are the key enzymes related to cartilage degradation in OA. The proteolytic cleavage of collagens and proteoglycans by MMPs are responsible for temporomandibular disorders [27, 28]. The present study has implicated that RES could inhibit MMP1 and MMP13 in MCC and increase the expression of cartilage markers ACAN and COL2 in vitro, which is one of the mechanism responsible for RES therapeutic effect.

In TMJOA mice, the structure of the condylar articular cartilage was destroyed significantly by injection in the upper cavity of TMJ compared to control mice. After RES treatment, histological evaluation of the cartilage tissue revealed a significantly reduced cartilage destruction compared to the OA group, which indicated the protective effects of resveratrol against MCC. Many in vivo studies demonstrated that RES may exert an antiosteoarthritic effect by inhibiting COX-2 gene expression and enzyme activity [29]. It was reported that RES decreased AGEs-stimulated expressions of MMP13 and prevented AGEs-mediated destruction of collagen II [30]. In TMJOA mice, our study found that RES could suppress the expression of COX-2, NF-*κ*B pathway, and MMPs and increase the expression of cartilage markers ACAN and COL2, which indicated that RES may be a promising agent in the treatment for TMJOA.

However, it should be realized that there are a few limitations of the experiments. More biological effects such as cell proliferation, more inflammatory mediators, and the mechanism through which COX-2 induced by inflammation were not demonstrated.

## 5. Conclusions

In conclusion, this study indicates the involvement of COX-2 on inflammation-induced condylar cartilage degeneration in temporomandibular osteoarthritis. The most intriguing aspect of this study is that the RES recovering MCC injury may be related to the inhibition of the expression of COX-2/NF-*κ*B. In addition, RES could increase the expressions of chondrogenic markers, suggesting that RES plays an important role in the remodeling of the cartilage in TMJOA.

## Figures and Tables

**Table 1:** The primer sequences for RT-PCR.

Gene	Sequences 5′-3′	PrimerBank ID
GAPDH	Forward: AGGTCGGTGTGAACGGATTTG Reverse: GGGGTCGTTGATGGCAACA	126012538c1
COX-2	Forward: TTCCAATCCATGTCAAAACCGT Reverse: AGTCCGGGTACAGTCACACTT	118130137c1
P65	Forward: GGGCTTGGAAATAGAGACATTGA Reverse: GTTCACGGATGACCTCTTTGTTT	20379991a1
MMP1	Forward: CCTTGATGAGACGTGGACCAA Reverse: ATGTGGTGTTGTTGCACCTGT	133778986c1
MMP13	Forward: TGTTTGCAGAGCACTACTTGAA Reverse: CAGTCACCTCTAAGCCAAAGAAA	291463259c1
ACAN	Forward: GTGGAGCCGTGTTTCCAAG Reverse: AGATGCTGTTGACTCGAACCT	116875857c1
COL2a	Forward: GGGTCACAGAGGTTACCCAG Reverse: ACCAGGGGAACCACTCTCAC	166064039c1

## Data Availability

Data used to support the findings of this study are available from the corresponding author upon request.
